# Technology Matters: A model for translating digital and data science innovations into Child and Adolescent Mental Health Services

**DOI:** 10.1111/camh.70003

**Published:** 2025-06-26

**Authors:** Alice Wickersham, William Bennett, Zoë Firth, Craig Colling, Jessica Penhallow, Johnny Downs, the CAMHS Digital Lab

**Affiliations:** ^1^ CAMHS Digital Lab, Department of Child and Adolescent Psychiatry Institute of Psychiatry, Psychology & Neuroscience, King's College London London UK; ^2^ South London and Maudsley NHS Foundation Trust London UK

**Keywords:** Service development, digital innovations, data science, large data, CAMHS

## Abstract

As digital mental health research and technology continue to grow, a systematic approach to quickly and safely translating digital innovation into Child and Adolescent Mental Health Services (CAMHS) is needed. Here, we provide an overview of the CAMHS Digital Lab, a service in London, United Kingdom, which integrates operational, clinical and research expertise to support digital discovery and translate research into practice. The service is organized into four workstreams: Population and clinical analytics; Data science and discovery; Digital therapeutics and assessment; and Education, outreach and training. This service provides a model for integrating digital innovation into mental health services which could be adopted elsewhere.

**Key Practitioner Message**

**What is known?**

- Digital and data science innovations present an opportunity to improve service delivery and meet growing demand, but there remains a significant gap between such advancements and their translation into clinical practice.

**What is new?**

- Services which bring together expertise in research, operations, and clinical work provide an opportunity to bridge this gap.

**What is significant for clinical practice?**

- Implementing such a model elsewhere would open opportunities for national and international collaboration and replication.

## Background

Child and Adolescent Mental Health Services (CAMHS) face growing demand, putting increasing pressure on services and resulting in extensive waiting lists. Services are in critical need of digital solutions, which quickly and safely improve delivery, from analytics which inform service resource allocation to delivery of evidence‐based digital interventions while patients await or step down from specialist mental health care. If used effectively, technology can also streamline administrative tasks to allow more time for patient contact. However, there remains a significant gap between advancements in digital health and data science and their translation into clinical care pathways.

## CAMHS Digital Lab

In this paper, we present a working service model based within the King's Maudsley Partnership—a collaboration between King's College London and South London and Maudsley NHS Foundation Trust—which could be adopted elsewhere as a University and healthcare provider partnership. The CAMHS Digital Lab is a team that brings together clinical, research, design, informatics, engineering and operational expertise to support the implementation of digital innovations in CAMHS serving local populations. It provides clinicians, service managers and commissioners with cost‐effective, high‐impact and design‐led initiatives to transform child mental health systems. Its objectives are to: (1) develop preventive strategies; (2) adapt existing treatments and make them more widely available; (3) enhance CAMHS delivery. The CAMHS Digital Lab is organised into four workstreams, as outlined below.

## Workstream 1: Population and clinical analytics

The CAMHS Digital Lab uses data collected by South London and Maudsley CAMHS and other public services to understand mental health needs, identify risk factors, highlight inequalities, and inform decision‐making by clinicians and service managers. The workstream's activities are overseen by the CAMHS Health Intelligence Group to ensure that work undertaken is directly relevant to service delivery.

One of the main data assets we draw from is the Clinical Record Interactive Search (CRIS), which provides research access to de‐identified electronic health records from South London and Maudsley's secondary mental health services, including CAMHS (Downs et al., 2019). We can use CRIS to better understand CAMHS treatment pathways—for example, examining time spent in services among young people with depression (work which we have also replicated in Cambridgeshire and Peterborough NHS Foundation Trust) (Wickersham et al., 2024).

Data from our local CAMHS are fed directly back to clinicians through clinical dashboards at both a caseload and individual patient level. Dashboards were designed and developed with NHS partners to provide easily accessible health intelligence tools at the point of care to assist in clinical decision making. This includes caseload management tools and longitudinal patient‐reported outcome measures (PROMs).

We have also linked our CRIS data to other external data sources, like the Department for Education's National Pupil Database, which comprises sociodemographic, education and social care data for all pupils in England's state‐maintained schools (Downs et al., 2019). Using this, we have enhanced our understanding of relationships between mental health and school absence, depression and educational attainment, and special educational needs and self‐harm. This work provides an evidence base for the UK Department for Education, Integrated Care Boards, and other NHS organisations that plan health services for local populations, local councils/authorities/districts, and liaison services supporting links between schools and CAMHS.

## Workstream 2: Digital therapeutics and assessment

The CAMHS Digital Lab delivers digital products to improve service delivery, clinical assessment and treatment. With significant and continual input from families, young people, clinicians and teachers, we have co‐developed myHealthE, a secure online portal for families who have been referred to CAMHS. Through the portal, families can access referring information, submit PROMs, and find mental health resources, advice and signposting through a virtual waiting room. These resources have been selected by local clinicians and service managers in a dedicated content management group. PROM completion rates through traditional pen‐and‐paper methods can be as low as 8%, but myHealthE is estimated to have increased local PROM completion rates 12‐fold (Morris et al., 2022).

We are also pioneering other novel data collection techniques to inform assessment and treatment. Our mood monitoring app, myJournE, was co‐designed with young people in South London (Grant, Widnall, Cross, Simonoff, & Downs, 2020). It is currently used by the Maudsley Education Consultation Service to help local schools track the mental health of their pupil body and inform service provision in their schools. We are also pioneering a child‐friendly wearable device and app as part of the Paediatric Actigraphic Clinical Evaluation project. The device was jointly designed by clinicians, researchers, designers, engineers, families and children with ADHD in order to track activity levels and monitor treatment response among children with ADHD (Morris et al., 2024).

The CAMHS Digital Lab also considers how new or existing digital technologies could be incorporated into CAMHS care pathways, such as digital health interventions. While the National Institute for Health and Care Excellence (NICE) provides recommendations for digital health tools, there is currently no standardised method for adopting them into clinical practice or reviewing and prioritising tools that meet local service needs. To overcome this, our Digital Therapies and Assessment Oversight Group comprises clinicians, informaticians, and researchers to oversee the discovery, development, and implementation of digital tools into local CAMHS, from digital parenting interventions (https://optimastudy.co.uk/) to self‐guided computerised cognitive behavioural therapy (https://digitalyouth.ac.uk/research/research‐projects/sparx‐developing‐precision‐digital‐cognitive‐behavioural‐therapy‐for‐young‐people‐with‐depression/).

## Workstream 3: Data science and discovery

As well as pioneering tools, we use novel data science methods to enhance our understanding of child and adolescent mental health. For example, one of the challenges of working with electronic patient records data via CRIS is the volume of unstructured clinical notes, which are a rich and nuanced source of information, but are difficult to analyse given their length, variability and complexity. To overcome this, we have developed Natural Language Processing (NLP) tools that extract information from large volumes of text. We have supported the development and validation of multiple NLP tools in young people's records to better identify causes and consequences of mental health problems, including self‐harm, bullying, online activity, treatment resistance and substance misuse.

We are also exploring how other new methods and types of data could inform clinical service delivery. For example, we have trialled machine learning prediction models using early childhood school data to predict the likelihood of meeting ADHD diagnostic criteria at the individual level, and automated tools applied to parent speech data to predict offspring mental health (Fisher, Firth, Aicardi, & Downs, 2024). This workstream allows us to remain responsive to new developments in digital health technology, developing and testing them for eventual application in clinical services.

## Workstream 4: Education, outreach and training

We prioritise engaging with local stakeholders to support the use and further development of our tools. We run workshops, consult with service users and practitioners on our research (Grant et al., 2020), and create materials to communicate our work to our partners and the public. The CAMHS Digital Lab runs more than 10 Patient and Public Involvement (PPI) sessions a year with a range of stakeholders, from children and young people to caregivers and clinicians. Our PPI sessions are innovative; we employ novel methods such as fictional storytelling in our workshops, and we recently published a novel framework for remote co‐design with and for neurodiverse children (Morris et al., 2024).

We supervise undergraduate, postgraduate, research and medical students to build digital expertise and capacity. We also share our expertise more widely with other clinical groups and researchers around the UK. We disseminate our work through traditional academic routes, such as peer‐reviewed journals, conferences, and book chapters (Wickersham & Downs, 2023). But we also take care to communicate our work through non‐academic channels to engage clinicians, policy‐makers and the public, for example, by producing explainer videos, blog posts, news articles, co‐production and storytelling (www.stephenoram.net/extractinghumanity/). This ensures that our digital innovations have a meaningful impact beyond academia and our local services (www.camhsdlab.co.uk).

## Discussion

The CAMHS Digital Lab provides a model for how integration between clinical, research, and operational groups can accelerate the translation of digital innovations into routine clinical practice. Other CAMHS across the UK and internationally could adopt a similar approach, or focus on specific elements to meet their local needs. For example, in services facing increasing demand and a lack of resources to provide timely face‐to‐face care, they might prioritise the implementation of an oversight group to review and systematically recommend existing digital health interventions to patients on waiting lists. Outside the UK, low‐ and middle‐income countries may particularly benefit from organising such groups to commission remotely deliverable tools in areas where child mental health services may be less readily available.

There is further opportunity for national and international collaboration and replication. For example, linked health and education data from across England is available from ECHILD (Education and Child Health Insights from Linked Data), offering the potential to scale our research and novel methods nationally. Similar data sources are also available in many other countries—for example, Scandinavian countries have historically led the field of population data linkage, such that greater collaboration between researchers could allow international replication to offer a more global perspective on child and adolescent mental health needs.

## Conclusion

As pressure on CAMHS continues to grow, services should look to digital solutions for improving health intelligence, informing service delivery and meeting local need. Establishing cross‐disciplinary teams bridging academia and clinical services can safely and effectively speed the translation of digital innovations into delivery and practice.

## Funding information

This paper from the CAMHS Digital Lab represents independent research part funded by the NIHR Maudsley Biomedical Research Centre, South London and Maudsley NHS Foundation Trust and King's College London. The views expressed are those of the author(s) and not necessarily those of the NHS, the NIHR or the Department of Health and Social Care.

## Conflicts of interest statement

All authors are members of the CAMHS Digital Lab.

## Author contributions

All named authors contributed to the manuscript drafting and/or review. All authors approve the manuscript for submission.

## Ethics statement

This article provides an overview of an existing service model and does not contain any research. Therefore, ethical approval is not required.

## Data Availability

Data sharing not applicable to this article as no datasets were generated or analysed during the current study.
